# A replaceable liposomal aptamer for the ultrasensitive and rapid detection of biotin

**DOI:** 10.1038/srep21369

**Published:** 2016-02-23

**Authors:** Tzu-Cheng Sung, Wen-Yih Chen, Pramod Shah, Chien-Sheng Chen

**Affiliations:** 1 Graduate Institute of Systems Biology and Bioinformatics, National Central University, Taoyuan City, 32001, Taiwan; 2 Department of Biomedical Sciences and Engineering, National Central University, Taoyuan City, 32001, Taiwan; 3 Department of Chemical and Materials Engineering, National Central University, Taoyuan City, 32001, Taiwan

## Abstract

Biotin is an essential vitamin which plays an important role for maintaining normal physiological function. A rapid, sensitive, and simple method is necessary to monitor the biotin level. Here, we reported a replacement assay for the detection of biotin using a replaceable liposomal aptamer. Replacement assay is a competitive assay where a sample analyte replaces the labeled competitor of analyte out of its biorecognition element on a surface. It is user friendly and time-saving because of washing free. We used aptamer as a competitor, not a biorecognition element as tradition. To label aptamers, we used cholesterol-conjugated aptamers to tag signal-amplifying-liposomes. Without the need of conjugation procedure, aptamers can be easily incorporated into the surface of dye-encapsulating liposomes. Two aptamers as competitors of biotin, ST-21 and ST-21M with different affinities to streptavidin, were studied in parallel for the detection of biotin using replacement assays. ST-21 and ST-21M aptamers reached to limits of detection of 1.32 pg/80 μl and 0.47 pg/80 μl, respectively. The dynamic ranges of our assays using ST-21 and ST-21M aptamers were seven and four orders of magnitude, respectively. This assay can be completed in 20 minutes without washing steps. These results were overall better than previous reported assays.

Biotin is an essential B-vitamin (Vitamin B7 or H), which is not synthesized by the human body. It plays an important role for amino acid metabolism, fatty acid synthesis and gluconeogenesis as an important cofactor of several carboxylases. Lack of biotin will lead to hair loss, conjunctivitis, brittle nail, red rash, weight loss, mental disorder and abnormal baby development^1^,^2^,^3^,^4^. It was also reported that biotin helps diabetic patients to maintain the blood sugar level^5^. Therefore, it is important to monitor the biotin level in human body, especially for pregnant women^2^, children^3^ and people with diabetes^5^.

Detection of biotin can be simply categorized into non-biosensing and biosensing methods. Non-biosensing methods include microbiological assay, UV spectroscopy-based methods, and several liquid chromatography (LC) methods^6^,^7^. Whereas, biosensing is a rapid, sensitive and simple method to detect analytes using biorecognition elements. Today, the most commonly used biorecognition elements for biotin detection are enzymes^8^, antibodies^9^,^10^,^11^ and streptavidin (or avidin)^8^,^12^. Streptavidin have been the most popular biorecognition elements for biotin detection because of their high sensitivity and selectivity.

Most of the biosensing methods used for biotin detection are regular competitive assays. The competitive assays are of two types: the one in which the competition is between sample biotin and labeled competitor biotin for the binding of immobilized biorecognition elements. The other is the competition between sample biotin and immobilized competitor biotin for the binding of labeled biorecognition elements. For example, isotope dilution assays is a competition assay between radioactively labeled and sample biotin for avidin binding^13^. Enzyme protein binding assay is a competition assay between sample biotin and immobilized biotin for the binding of avidin linked horse-radish peroxidase (HRP)^8^. Electrochemical magneto biosensing assay, is a competition assay between HRP-labeled biotin and sample biotin for the binding of streptavidin on magnetic microbeads^14^. Ho and her colleagues^9^,^10^,^15^ have reported various competition assays for biotin dectection where competition is between sample biotin and biotin-tagged liposome for the binding of anti-biotin antibody. These methods include flow injection analysis with a liposome-amplified competitive bioluminescence immunoassay^9^, immunoaffinity chromatographic assay with a liposomal fluorescent biolabel^10^ and nano-structured material plated screen-printed carbon electrode^15^. All these methods used liposome to label competitor biotin with the signal amplification function.

Liposomes can encapsulate hundreds of thousands of bioluminescence, fluorescent dyes or other signals which provides strong signal amplification. This makes liposomes very sensitive in biosensing assays. For the same reason, in this study, we also used liposomes as the signal amplification system and competitor labeling. However, instead of a regular competitive format, we here used replacement format for the detection of biotin. A replacement assay is also a competitive assay where a sample analyte replaces the labeled competitor of analyte out of its biorecognition element on a surface^16^. The great advantage of the replacement assay is that no reagent addition or wash steps are required after loading samples because the signal is directly from the released labeled competitors. Therefore, the assay can be user-friendly and the assay time is reduced. Furthermore, the signal can increase with the increasing of analyte concentration, which is highly desirable compared to a regular competitive assay^17^.

An effective replacement assay requires low affinity between the biorecognition element and the competitor of analyte in a way such that analyte is able to replace the competitior. However, it is not easy to have a competitor with lower affinity than the analyte, and is still able to bind to its biorecognition element. In this study, we proposed to use aptamer as a competitor of replacement assays. An aptamer is an oligonucleotide that binds to an analyte without base pairing^18^. Aptamers are usually screened from a large random nucleic acid sequence pool, which is called systematic evolution of ligands by exponential enrichment (SELEX)^19^. Since the affinity of most screened aptamers are not high in the first few rounds of SELEX, aptamers will be an ideal competitor of analyte in replacement biosensing assays. Any aptamer as a competitor can be easily screened with SELEX by a known analyte-binding ligand. To label the aptamer competitor with liposome, we used a conjugation-free procedure to form a liposomal aptamer as a labeled competitor for the replacement assay. Aptamer can be easily tagged with cholesterol during the oligonucleotide synthesis, and the cholesterol-tagged aptamers can be easily incorporated into liposomes by simply mixing them with other lipids during the liposome fabrication procedure. Here, we chose two aptamers as biotin competitors: ST-21M and ST-21, which specifically bound to biotin-binding site of streptavidin, not even biotin^20^.

In this study, we developed a 96-well plate assay for the detection of biotin. This replaceable liposomal aptamer formed complex with streptavidin which were immobilized on 96-well plate. Sample biotin replaced the liposomal aptamer from the immobilized streptavidin without any washing steps. This liposomal aptamer provide not only an ultrasensitive and rapid detection of biotin, but also an alternative use of aptamer in the field of biosensing.

## Results

### Fabrication of liposomal aptamers

**Figure 1:**
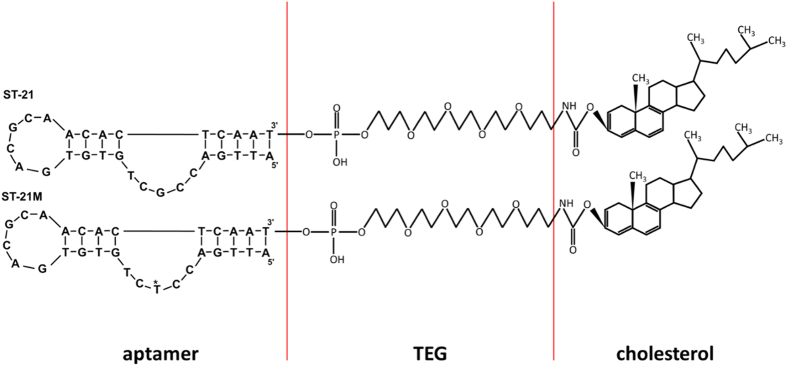
The streptavidin-binding aptamers, ST-21 and ST-21M, with the modification of TEG and cholesterol. TEG spacer provides an easier approach for streptavidin-binding. Cholesterol was used to incorporate into lipid bilayer of liposomes. The asterisk indicates mutation site in the sequence of ST-21M.

**Figure 2:**
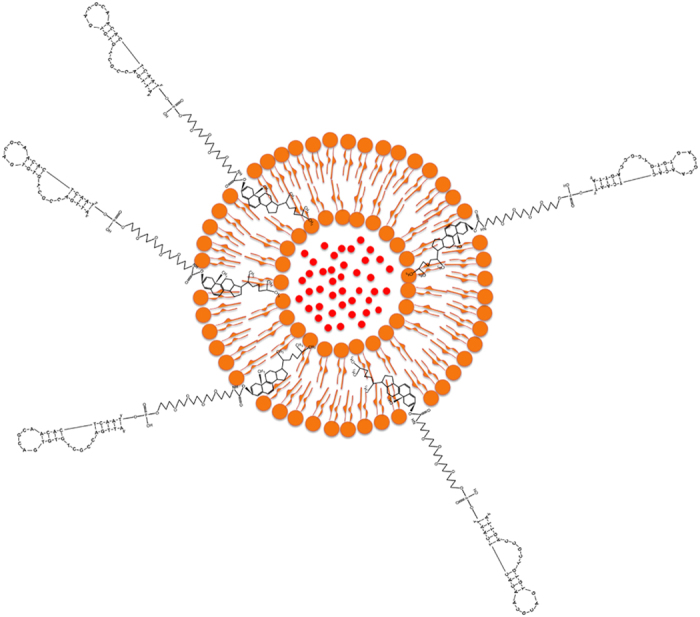
The liposomal aptamer with the ability of binding to streptavidin. The cholesterol incorporated into lipid bilayer through hydrophobic interactions. The liposomes encapsulated hundreds of thousands of fluorescent dyes for providing strong signal to aptamer.

Two aptamers, ST-21and ST-21M, with the binding ability to streptavidin were chosen for preparation of liposomal aptamers. The sequences of the ST-21 and ST-21M aptamers were 5′-ATTGACCGCTGTGTGACGCAACACTCAAT-3′ and 5′-ATTGACCTCTGTGTGACGCAACACTCAA T-3′, respectively. They were designed with 1 base difference (from 5′ the 8^th^ G is replaced by T) and their dissociation constant were 92.85 nM^21^ and 3870 nM, respectively. Each aptamer was modified to have 3′- cholesterol (Fig. 1), which was incorporated into the lipid bilayer of liposomal nanovesicles (Fig. 2). TEG spacer was included to provide flexibility for easier approach to bind streptavidin. The cholesterol provides an easy way to tag aptamers on the surface of liposomes during liposome preparation using lipid hydration method. Compared to a laborious and time-consuming bioconjugation process, this cholesterol modified aptamer is able to tag on the liposome surface directly in the liposome fabrication procedure without the need of bioconjugation. The aptamers were mixed with lipid mixture in a molar percentage of 18.42% DPPG, 35.52% DPPC, and 45.93% cholesterol in organic solvent. The organic solvent was removed by nitrogen to yield a thin lipid film. High concentration SRB encapsulant was then added to hydrate the lipid mixture. The preparation of liposomal aptamers in a similar size range is a key to develop a reliable replacement platform. Extrusion of liposomes through a polycarbonate membrane filter helps to uniform the liposome size. The liposomes were extruded 30 times through 200 nm filters and the final average size was determined by a DLS assay. The average size of ST-21 and ST-21M liposomal aptamers were 151 ± 37 and 138 ± 43 nm, respectively (Figure S3). The stock concentrations of ST-21 and ST-21M liposomal aptamers are 2.43×10^7^ and 2.68×10^7^ liposomes/ml, respectively (Figure S4), which were determined by flow cytometry. We also checked the stability of liposomes. After being kept at 4 °C after three months, these liposomal aptamers still retained its binding ability to streptavidin. This suggested that the liposomal aptamers were very stable.

### Streptavidin-binding test of liposomal aptamers

**Figure 3:**
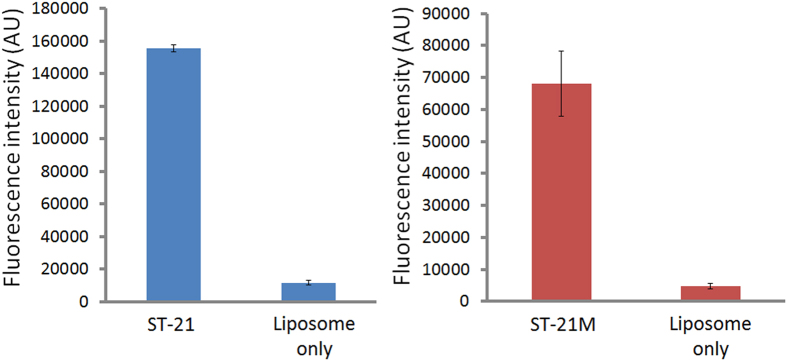
Streptavidin binding test of liposomal aptamer ST-21 (**A**) and ST-21M (**B**). The Liposome only is negative control without aptamer-tagged. Both liposomal aptamers showed strong binding to streptavidin, indicating the successful fabrication of liposomal aptamers.

To test if we really successfully fabricated ST-21 and ST-21M liposomal aptamers, we added the liposomal aptamers on streptavidin-coated plates and observed the binding ability between liposomal aptamers and streptavidin. After adding liposomal aptamers for one hour incubation, unbound liposomal aptamers were removed by several washes. Because our ST-21 and ST-21M are streptavidin binding aptamers, the liposomal aptamers would bind to streptavidin. Figure 3 shows the binding ability of two liposomal aptamers to streptavidin. Compared to the negative control (liposomes without aptamer tag, liposomes only in fig. 3), both liposomal aptamers had at least 10 fold signals (Fig. 3), indicating that both aptamers still preserved binding ability to streptavidin after incorporated into liposomes. This suggests that liposomal aptamers were successfully fabricated by a hydration method and can be used for replacement assays.

### Optimization of replacement time

**Figure 4:**
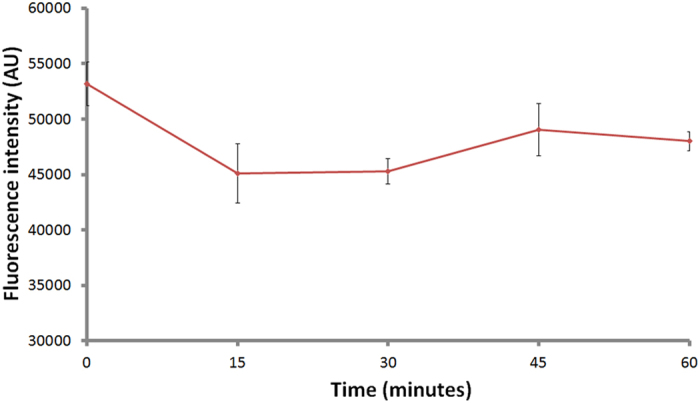
Optimization of replacement time. The fluorescent signal of liposomal aptamer ST-21 decreased after addition of biotin till 15 minute incubation. More than 15 minute replacement time shows no significant decrease.

At this step, we were curious about the incubation time of biotin for effective replacement. Therefore, we developed an assay to study the incubation time after addition of biotin. First, we formed the complexes of streptavidin and liposomal aptamers on the plate, and then we added biotin to replace liposomal aptamers. Because biotin has lower dissociation constants (Kd) than aptamer, it can replace the binding of liposomal aptamers to streptavidin. In this assay, replaced liposomal aptamers were removed by washing. Then 30 mM of n-OG was added into wells to release SRB from the un-replaced liposomal aptamers. The fluorescence of SRB was measured by a 96-well plate reader. Since higher biotin concentration could have higher replacement efficiency and requires less incubation time for replacement, we chose a low biotin concentration (15 pg/ml) to optimize the incubation time for replacement. Figure 4 shows that fluorescent signals of liposomal aptamers ST-21 decrease dramatically after addition of biotin till 15 minute incubation. More than 15 minute replacement time shows no significant signal decreasing. This indicates that biotin cannot replace more liposomal aptamers after 15 minutes incubation. Therefore, we chose to incubate the biotin sample for 15 minutes for dose-response assays in this study.

### Dose response of replacement assays for the detection of biotin

**Figure 5:**
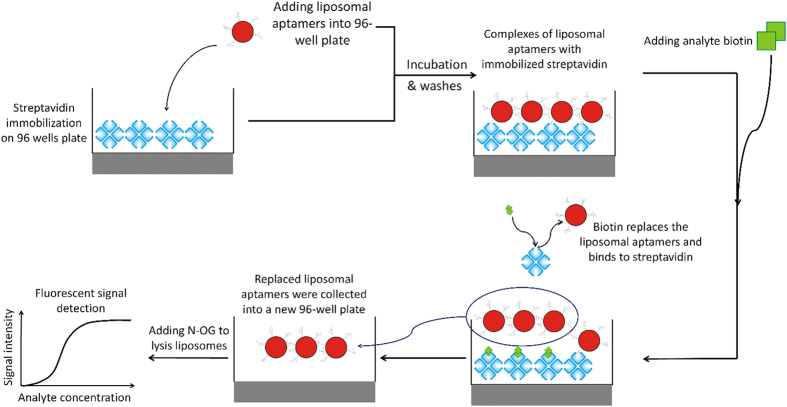
Schematic diagram of our liposomal aptamer replacement assay for the detection of biotin. The liposomal aptamers were pre-bound to streptavidin coated on a 96-well plate. When biotin was presented in the sample, the liposomal aptamers will be replaced by biotin. Fluorescent signals were measured by a microtiter plate reader.

**Figure 6:**
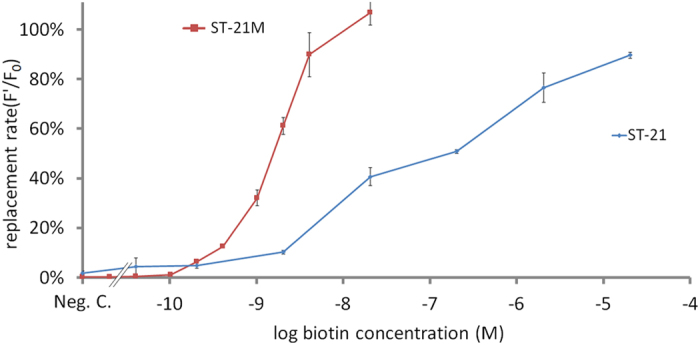
Dose response curves of biotin detection using liposomal aptamers (ST-21 and ST-21M). The replacement rates increase with the increasing of biotin concentration. ST-21M shows steeper slope than ST-21. Error bars represent the standard deviations of triplicate measurements.

Dose response curves reveal the dynamic range, sensitivity, and detection limit of a biosensing assay. Therefore, the dose response of replacement assays for the detection of biotin was evaluated in our proposed replacement assay. Figure 5 shows the design of the replacement assay for the detection of biotin. A 96-well plate was first coated with the complexes of streptavidin and liposomal aptamers. Different concentrations of biotin samples were added to the plate wells. When the biotin is presented in the sample, the dissociation between liposomal aptamers and coated streptavidin occurred due to the replacement of biotin. Afterwards, we collected replaced liposomal aptamers in the solution, and then lysed the liposomes with n-OG to detect fluorescence released from liposomes. The fluorescent signal was then measured by a microtiter plate reader. For the comparison of the dynamic range, sensitivity, and limit of detection (LOD) between the two liposomal aptamers, we normalized the fluorescent signals. (F’) by the comparison with the fluorescence of all un-replaced liposomes on the well of the negative control (F_0_) using the equation of replacement rate = (F’/F_0_)×100%. Because the negative control has no biotin to replace liposomal aptamers on the plate well surface, the un-replaced fluorescence on the well of the negative control represents the overall liposomal aptamers on the plate well. Therefore, this ratio represents the replacement rate of each biotin sample. We used this replacement rate as the Y-axis of the biotin dose response curve. Figure 6 shows the biotin dose response curves for both ST-21 and ST-21M aptamers. The result showed that the slope of ST-21M dose response is steeper than ST-21, indicating that ST-21M is more sensitive than ST-21 liposomal aptamers. The LOD was defined as the lowest concentration of analyte producing a fluorescence intensity that is three times of standard deviations higher than the mean intensity at zero concentration (negative control). According to this definition, the LOD for liposomal aptamers ST-21and ST-21M were 6.75×10^−11^ M (1.32 pg/80 μl) and 2.39×10^−11^ M (0.47 pg/80 μl), respectively. ST-21M shows both better sensitivity and LOD than ST-21. These results were expected because the Kds of ST-21 and ST-21M are 92.85 nM and 3870 nM, respectively. The Kd of ST-21M is near 42 folds larger than ST-21, which makes ST-21M is easier to be replaced by biotin than ST-21.

We also observed the dynamic range of the dose response curve. The dynamic range defined as the biotin quantitation ranges from LOD to the saturated concentration of biotin replacement. Compared with these 2 liposomal aptamers, ST-21 had a dynamic range of seven orders of magnitude (6.75×10^−11^ to 2.04×10^−4^ M); and ST-21M had over four orders of magnitude (2.39×10^−11^ to 2.04×10^−7^ M). ST-21 liposomal aptamer has a wider dynamic range. Note these findings are consistent with the previous study by Gooding and his colleagues^22^. They reported that a higher affinity competitor of analyte were able to form more complexes with the biorecognition element; thereby allowing more analyte to replace the competitors. Therefore, our result can be explained by that there are more ST-21 complexes with streptavidin; thereby allowing more biotin to replace ST-21, and thus the dynamic range of ST-21 is wider than ST-21M. Another possible reason is that the higher affinity ST-21 were not easily replaced, allowing higher concentration of biotin to replace. Therefore, higher affinity competitors have a wider dynamic range.

## Discussion

In 1990, the discovery of aptamers opened a new era for biosensing technology^18^,^19^,^23^. Aptamers were used as biorecognition elements to detect metal ions, organic compounds, proteins or other biomolecules, virus and cells^17^. In this study, we reported a strategy to use aptamer as a competitor instead of a biorecognition element in replacement assays. Replacement assays have many advantages. In addition to simple and time-saving procedures without the need of washes, the dose response pattern of a replacement assay is more desirable compared to a regular competitive assay. Biosensing assay determines the LOD based on a dose response curve and LOD is defined as the lowest concentration of analyte producing a fluorescence intensity that is three standard deviations (SDs) higher or lower than the mean intensity at zero concentration (negative control). However, for a regular competitive assay, the negative control has the highest signal intensity and thus has the largest SD. According to the LOD definition, this large SD in the negative control causes the large LOD after calculations. On the contrary, in replacement assay, the signal increases with the increasing analyte concentration. Thus, negative control has the lowest signal intensity and thus has the smallest SD. Therefore, a replacement assay usually has a lower LOD compared to a regular competitive assay. Although a replacement assay is highly favorable, the application of replacement assay is limited because it is not easy to fabricate a competitor with a lower affinity to the biorecognition elements, especially for a small molecule, such as biotin. Also aptamer can be easily screened with SELEX by a known analyte-binding ligand. Therefore, this replaceable liposomal aptamer strategy can be applied on the detection of other target analytes. Normally, it is also troublesome to label a competitor. Here, we also provided an easy method to label aptamers with a signal amplification system (liposome). The cholesterol modification provides an incorporation site in the lipid bilayer during liposome formation. Therefore, no conjugation process is ne eded for labeling. Liposomes are widely used in biosensing as signal amplifying reagents due to their high surface area and internal volume^24^. Previously, we developed protein G-liposomes as universal reagents for immunoassays. They have been used in an immunomagnetic bead sandwich assays for the detection of *Escherichia coli* O157:H7^25^ and have also been demonstrated in multiplexed immunosorbent assays for the simultaneous detection of *E*. *coli* O157:H7, *Salmonella*, and *Listeria monocytogenes*^26^. Many other reports showed that liposomes provided ultra sensitivity in biosensing assays^9^,^10^. Combination of both the advantages of aptamer and liposomes, the liposomal aptamer is an excellent competitor in a replacement assay.

In order to assure the reproducibility and stability of the liposomal aptamer for replacement assay in this study. The protocol of our liposomal aptamer fabrications was established and standardized. We controlled the liposome hydration temperature at 60 °C to make sure that all the lipids and lipid-tagged aptamers would be all self-assembled and transform to liposomes. Therefore, the aptamer percentage (0.13%) we added can be the same as the aptamer percentage in the liposomes. In this way, the aptamer amount in each liposome was controlled. We also extruded liposomes through 200 nm polycarbonate filter 30 times to unify the liposomal aptamer size, and removed un-encapsulated dye to make sure all the dyes are inside the aptamer-tagged liposomes. The encapsulated solution volume was controlled by the size through the filter extrusion and the dye concentration would not be changed after being encapsulated into liposomes; therefore, the quantity of dye molecules contained in each liposome was controlled. The controlled quality of liposomal aptamer helped us to establish a reproducible and reliable platform for replaceable liposomal aptamer assay.

We compared the LODs of both liposomal aptamers with other biotin assays (Table 1). ST-21M showed the lowest LOD (5.83 pg/ml) in all biotin detection assays. Even ST-21 (16.48 pg/ml) is also comparable with others. For comparison between our two aptamers, the affinity of ST-21 to streptavidin is much higher (near 42 folds) than ST-21M; however, the LOD of ST-21 is only a little bit larger than ST-21M, indicating that the LOD was not much affected by the aptamer affinity as long as the aptamer is able to be replaced by the analyte.

## Methods

### General materials

Biotin, streptavidin, bovine serum albumin, Tween-20, tris base, sodium chloride, cholesterol, chloroform, sulforhodamine B (SRB), noctyl-beta-d-glucopyranoside (n-OG) and sucrose were purchased from Sigma-Aldrich. Dipalmitoylphosphatidylcholine (DPPC), dipalmotoylphosphatidylglycerol (DPPG) and the liposome extruder were purchased from Avanti Polar Lipids. Sepharose CL4B was purchased from GE healthcare. Maxisorp 96-well black plate, 200 nm filter membranes were purchased from Whatman. Aptamers are synthesized by IDT (Integrated DNA Technologies, USA). Two aptamers (ST-21: 5′-ATTGACC**G**CTGT GTGACGCAACACTCAAT-3′ and ST-21M: 5′-ATTGACC**T**CTGTGTGACGCAA CACTCAAT-3′) were used in this study.

### Preparation of liposomal aptamers

The quality of aptamers was checked by HPLC and MS assay (Figs S1 and S2). The liposomal aptamers were prepared using the lipid hydration method from lipid mixture with some modifications^25^. The lipid mixture consisted of 18.42% DPPG, 35.52% DPPC, 45.93% cholesterol and 0.13% aptamer (ST-21 or ST-21M). The lipid mixture was dissolved in a mixture of chloroform and methanol (3:1 volume ratio). The organic solvent was evaporated using compressed nitrogen gas, and a thin lipid film was thus generated. The lipid film was then hydrated by 150 mM of SRB solution at 60 °C for 45 minutes. After being fully hydrated, the liposomal aptamers were extruded 30 times through a 200 nm polycarbonate filter membrane and then purified by gel filtration chromatography using Sepharose CL-4B. The sizes of liposomal aptamers were determined by dynamic light scattering (DLS) using nano partica SZ-100 instrument (Horiba, Japan). Whereas, the concentration of liposomal aptamers was determined by flow cytometry with Muse cell analyzer (Merck Millipore, Germany) using 1ml liposomal aptamer solution (10 μl stock liposomal aptamers with 990 μl TBS-sucrose, 100 folds dilution).

### Test of streptavidin-binding ability of liposomal aptamers

The binding test was initiated by coating streptavidin (10 μg/ml) on a 96-well plate at room temperature (RT) for 1h. The streptavidin serves as a capture agent for liposomal aptamers. After streptavidin coating, the wells were blocked at RT for 1 hr with 0.01 mg/ml salmon sperm DNA and 1% BSA in TBS. After removing the excess blocking solution, 80 μl of liposomal aptamers were added into a 96-well plate. After 1 hr incubation at RT, unbound liposomal aptamers were removed and the wells were washed three times with TBS-including appropriates sucrose (TBS-sucrose) for osmolarity. Finally, 80 μl of 30 mM n-OG were added into the 96-well plate to rupture liposomes and release SRB. The fluorescent signal was measured by a 96-well plate reader (Synergy 2, BioTek) at excitation wavelength of 540 nm and emission wavelength of 590 nm.

### Optimization of replacement time

Liposomal aptamers were first incubated in streptavidin-coated plates for 1 hr at RT. After washing unbound liposomal aptamers, 80 μl of 15 pg/ml of biotin was then added to each well to replace liposomal aptamers, which were bound to streptavidin on the plate. The biotin incubation times we tested are 5, 10, 15, 30, and 60 minutes, respectively. After incubation at RT, replaced liposomal aptamers were removed and the wells were washed three times with TBS-sucrose. Finally, 80 μl of 30 mM n-OG was added into each well of the 96-well plate to rupture liposomes and release SRB. A 96-well plate reader was used to detect SRB at excitation wavelength of 540 nm and emission wavelength of 590 nm.

### Dose response of the replacement assay

We first incubated liposomal aptamers with streptavidin coated plates. After washing unbound liposomal aptamers, 80 μl of different concentrations of biotin were added to each well. After 15 minute incubation at RT, 60 μl of biotin-replaced liposomal aptamers solution in each well was transferred to a new 96-well plate. Finally, 6 μl of 300 mM n-OG was added into the new 96-well plate to rupture liposomes and release SRB. The fluorescence of SRB was measured at excitation wavelength of 540 nm and emission wavelength of 590 nm.

The SRB fluorescent signal of all liposomes bound to streptavidin on the well of negative control is defined as F0 for data calculation and normalization. The signal of biotin-replaced liposomal aptamers solution was defined as F’. All data were normalization as replacement rates followed by the equation: replacement rate = (F’/F_0_)×100%.

## Additional Information

**How to cite this article**: Sung, T.-C. *et al.* A replaceable liposomal aptamer for the ultrasensitive and rapid detection of biotin. *Sci. Rep.* **6**, 21369; doi: 10.1038/srep21369 (2016).

## Supplementary Material

Supplementary Information

## Figures and Tables

**Table 1:** Comparisons of this study to several reported biotin detection methods.

Detection methods	LOD	references
Aptamer as a competitor	ST-21 16.48 pg/ml	this study
	ST-21M 5.83 pg/ml	
ELISA	977 pg/ml	8
Electrochemical immunosensor	2.02 ng/ml	15
Electrochemical magneto	20 ng/ml	14
LC	1–9 ng/g	7
HPLC/MS	2.5 ng/ml	27
Flow injection liposome immunoanalytical system	250 pg/ml	9
Immunoaffinity chromatographic	10 pg/ml	10
Isotope dilution	1 ng/ ml	13
Kinetic spectrophotometry	180 ng/ml	28
Surface plasmon resonance	0.5 ng/ml	11
