# Supplementary material for: A replaceable liposomal aptamer for the ultrasensitive and rapid detection of biotin
Source: Sci Rep. 2016 Feb 23;6:21369. doi: 10.1038/srep21369 (PMC4763257; doi:10.1038/srep21369)
Supplement: Supplementary Information [file srep21369-s1.doc]

**A replaceable liposomal aptamer for the ultrasensitive and rapid detection of biotin**

# Tzu-Cheng Sung^1,2^, Wen-Yih Chen^3^, Pramod Shah^1,2^, and Chien-Sheng Chen^1,2,*^

Supplementary figures


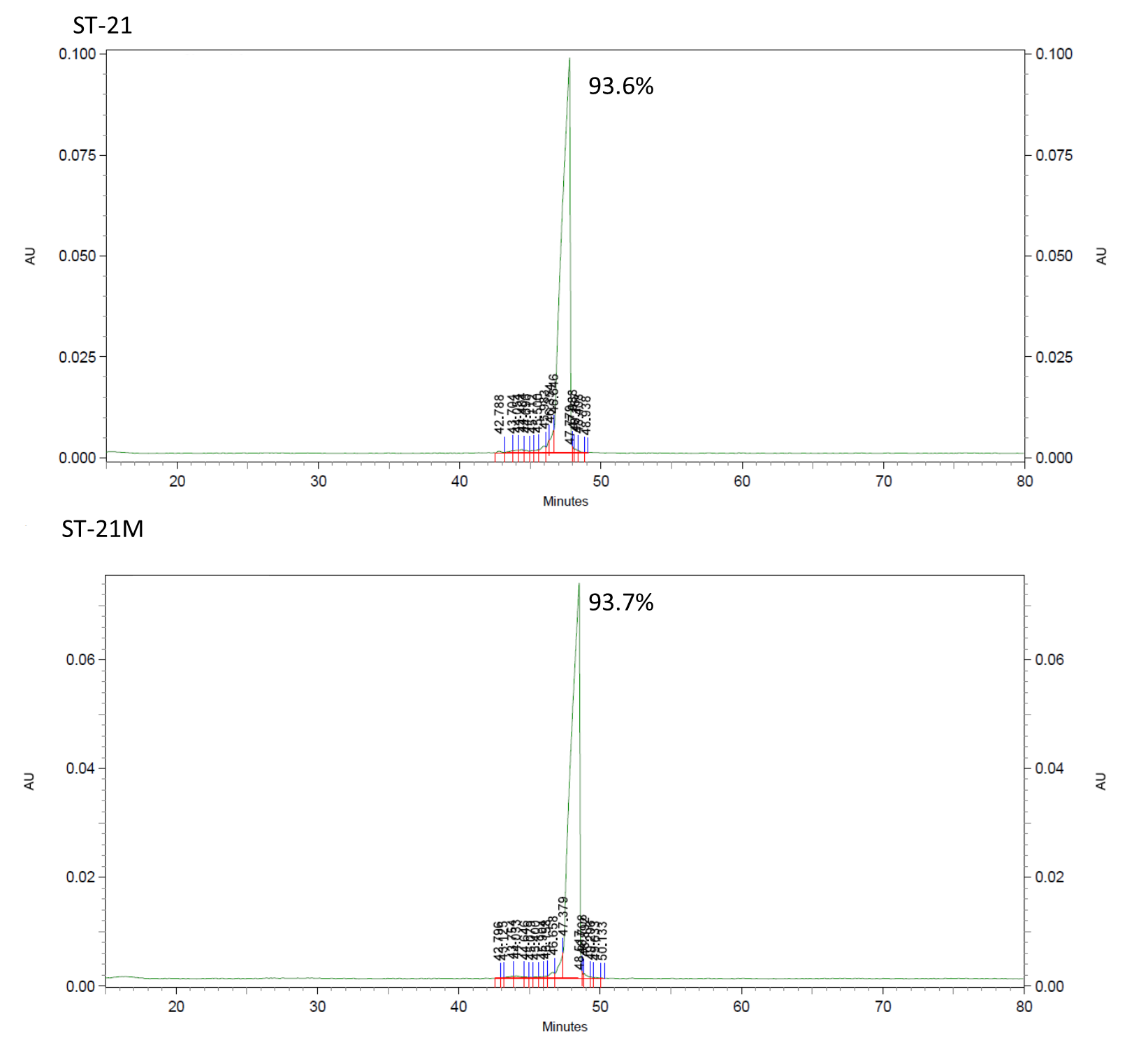


**Supplementary Figure S1.** HPLC chromatograms of ST-21 and ST-21M aptamers.


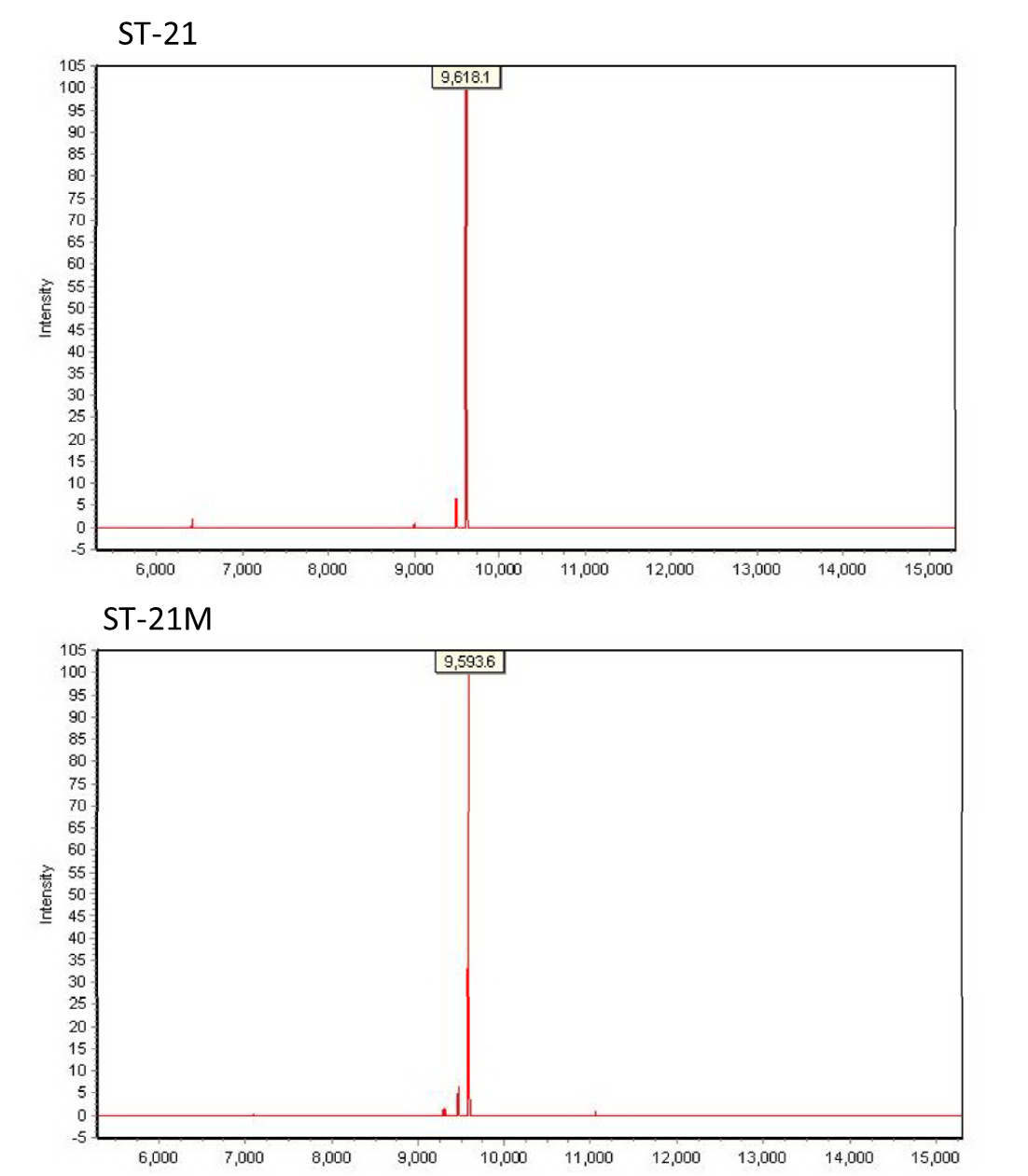


**Supplementary Figure S2.** Mass determination of ST-21 and ST-21M aptamers. The molecular mass shows on major peak of ST-21 and ST-21M (9618.1 and 9593.6, respectively) are close to theoretical molecular mass (9617.8 and 9592.8, respectively), Indicating the syntheses mass of ST-21 and ST-21M are correct.

**Supplementary Figure S3.** Size distribution of ST-21 and ST-21M liposomal aptamers. The average size of ST-21 and ST-21M liposomal aptamers are 151 ± 37 nm and 138± 43 nm, respectively.


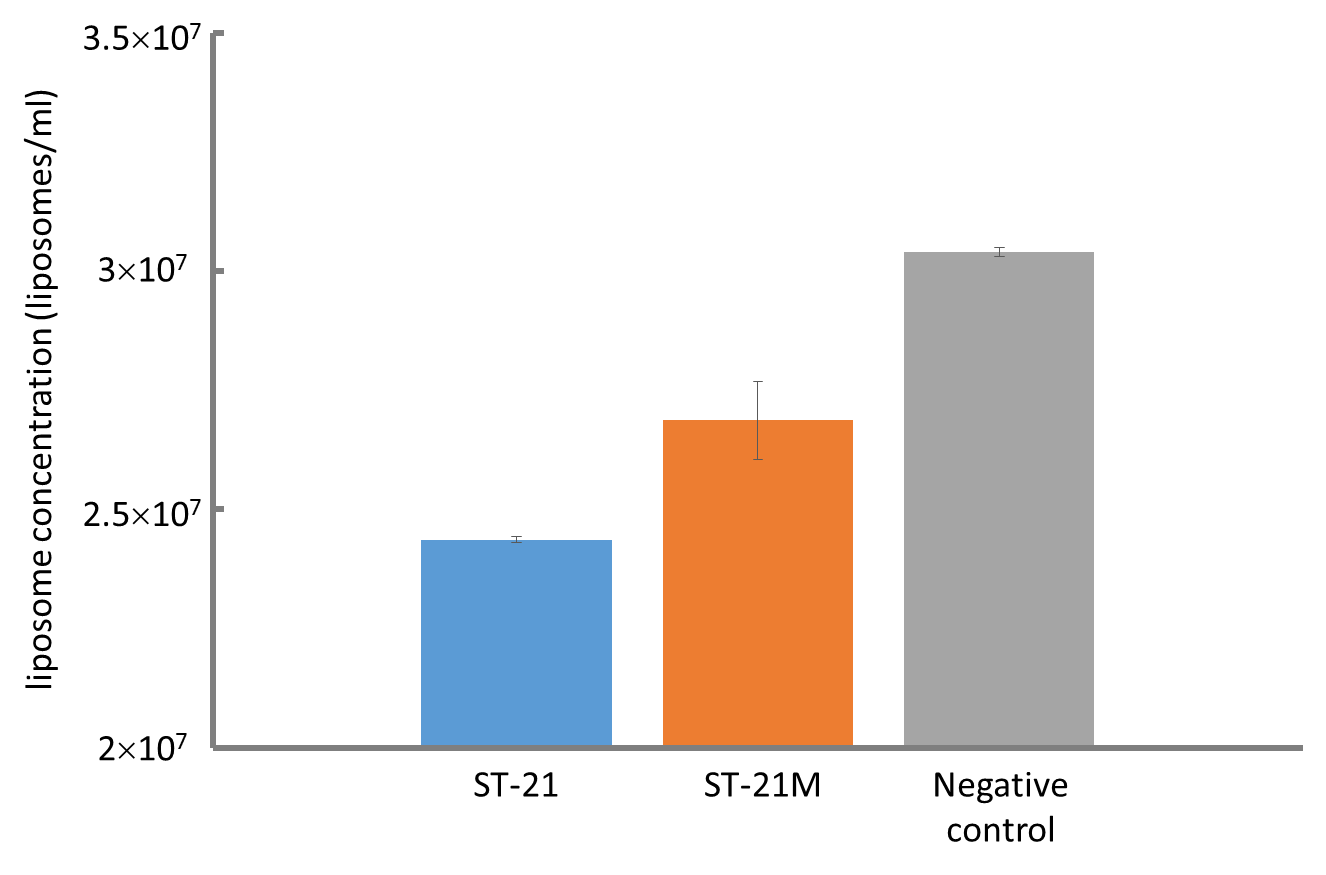


**Supplementary Figure S4.** Liposomal aptamers (ST-21 and ST-21M) and Negative control (liposomes without aptamer-tagged) concentration determined by flow cytometry. The concentration of ST-21 and ST-21M are 2.43×10^7^ and 2.68×10^7^ liposomes/ml, respectively. The Error bars represent the standard deviations of triplicate measurements.
